# Types of Ethical Problems and Expertise in Clinical Ethics Consultation in Psychiatry – Insights From a Qualitative Empirical Ethics Study

**DOI:** 10.3389/fpsyt.2021.558795

**Published:** 2021-05-25

**Authors:** Joschka Haltaufderheide, Jakov Gather, Georg Juckel, Jan Schildmann, Jochen Vollmann

**Affiliations:** ^1^Institute for Medical Ethics and History of Medicine, Ruhr University, Bochum, Germany; ^2^Department of Psychiatry, Psychotherapy, and Preventive Medicine, LWL University Hospital, Ruhr University, Bochum, Germany; ^3^Institute for History and Ethics of Medicine, Interdisciplinary Center for Health Sciences, Martin Luther University Halle-Wittenberg, Halle (Saale), Germany

**Keywords:** mental healthcare, clinical ethics support services, qualitative interviews, nonparticipant observation, ethical expertise

## Abstract

**Background:** Ethics consultation has been advocated as a valuable tool in ethically challenging clinical situations in healthcare. It is paramount for the development and implementation of clinical ethics support services (CESS) in psychiatry that interventions can address the moral needs of mental health professionals adequately and communicate the nature of the services clearly. This study explores types of ethical problems and concepts of ethical expertise as core elements of CESS in mental healthcare with the aim of contributing to the further development of ethical support in psychiatry.

**Methods:** We conducted 13 semi-structured interviews with mental health professionals and CESS members and triangulated them with four non-participant observations of ethical case consultations in psychiatry. Data were analyzed according to principles of grounded theory and are discussed from a normative perspective.

**Results:** The analysis of the empirical data reveals a typology of three different ethical problems professionals want to refer to CESS: (1) Dyadic problems based on the relationship between patients and professionals, (2) triangular problems, where a third party is involved and affected as a side effect, and (3) problems on a systemic level. However, CESS members focus largely on types (1) and (2), while the third remains unrecognized or members do not feel responsible for these problems. Furthermore, they reflect a strong inner tension connected to their role as ethical experts which can be depicted as a dilemma. On the one hand, as ethically trained people, they reject the idea that their judgments have expert status. On the other hand, they feel that mental health professionals reach out for them to obtain guidance and that it is their responsibility to offer it.

**Conclusion:** CESS members and professionals in mental healthcare have different ideas of the scope of responsibility of CESS. This contains the risk of misunderstandings and misconceptions and may affect the quality of consultations. It is necessary to adapt concepts of problem solving to practitioners' needs to overcome these problems. Secondly, CESS members struggle with their role as ethical experts. CESS members in psychiatry need to develop a stable professional identity. Theoretical clarification and practical training are needed.

## Introduction

Ethics consultation has been advocated as a valuable tool in ethically challenging situations in various healthcare settings in recent years (1–6). The development of clinical ethics support services (CESS) in Germany is supported by various medical bodies (7, 8) and sometimes even on a legal basis (39). The CESS are now widely implemented in somatic medicine in many western countries and have become an accepted tool to improve patient care (9–14).

It has often been suggested that CESS are less developed in psychiatric settings than in somatic care. Different medical cultures (e.g., lower hierarchies) and needs in consulting and competencies, such as the more communication-oriented attitude of mental health professionals, were assumed to be the reason (15–17). However, recent surveys revealed that more than half of the psychiatric hospitals in Germany, in fact, offer some kind of CESS which often includes ethical case consultation (18–20). Notwithstanding this fact, data indicate a mismatch in supply and demand since many of the hospitals reported ethical consultations in only a very few or even no cases per year. Despite the existence of CESS interventions, frequent occurrence of ethical issues and clinical routines demanding high ethical standards (21), ethical problems often seem to be discussed implicitly or elsewhere, such as in interdisciplinary team meetings or during supervision (18, 22, 23).

In addition to organizational and structural challenges, such as lack of resources or support (19), the successful implementation of CESS in psychiatry seems to hinge, *inter alia*, on two important challenges. Firstly, interventions must be able to respond adequately to the moral distress of mental health professionals (16). Moral distress is a psychological response which includes the experience of suffering (e.g., from anxiety, fear or anguish) connected to moral dilemmas, uncertainty or certainty accompanied by constraints (24, 25). Successful CESS, therefore, requires an understanding of the nature of an incoming request for support and the ability to tailor interventions to the needs of mental health professionals and the specific type of problem. Secondly, CESS members need to develop a professional role as ethicists on an equal footing with other professional roles in healthcare settings. This would allow the delimitation of their service from other interventions and to display their range of expertise and the value of ethical support clearly (16). However, very little is known about the ethical problems mental health professionals want to refer to CESS and how professional ethicist roles should be developed.

Against this background, this empirical ethics study investigates, at first, the types of ethical problems mental health professionals want to refer to CESS. It then explores assumptions about the professional roles of CESS members in mental healthcare settings. In a final step, the study's empirical data are analyzed from a normative perspective. The study aims, firstly, at improving the understanding of the needs for and expectations of mental health professionals regarding ethical advice in clinical psychiatry. Secondly, it aims at gaining an in-depth understanding of the underlying concepts and challenges in developing professional identities and ethical expertise as clinical ethicists based on the views of CESS members. Finally, different starting points for the promotion of CESS in psychiatry and the improvement of existing CESS in mental healthcare institutions are identified and discussed.

## Materials and Methods

### Theoretical Considerations

We hypothesized that learning more about the ethical problems referred to CESS by mental health professionals in the form of a typology provides a way of gaining a deeper understanding of the underlying needs and expectations. We adopted a narrow approach to defining “ethical problems” to ground this typology theoretically. According to this, an ethical problem can be determined by two propositions: Firstly, ethical problems are based on a relationship between a bearer of moral rights and an addressee of a claim. Secondly, in terms of content, ethical problems can be characterized by uncertainty regarding an ethically acceptable course of action or inability to carry out an accepted course. This can, for example, be the case when basic principles are in conflict or it is unclear which moral principle to apply (22, 26).

Regarding the professional role of an ethicist in CESS, we hypothesized that “ethical expertise” constitutes the core of this role. Ethical expertise can be generally described as a property of a person or group, consisting of certain skills, knowledge or both. Ethical expertise enables its carriers to exercise ethical considerations with a certain quality, legitimacy or authority (27). It, therefore, denotes a domain of expertise and a standard to distinguish experts from non-experts, resulting in a good reason to pay special attention to the advice of those fulfilling these standards (28, 29).

### Data Collection

We chose an explorative qualitative approach triangulating different data sources (30). Triangulation of different data sources can be used to mitigate bias and increase saturation. It is especially suitable for novel research questions and exploratory studies in which small sample sizes are to be expected and little is known about the phenomena in question. Data were collected from interviews with mental health professionals and CESS members. In addition, we conducted non-participant observations of ethical case consultations in different mental healthcare institutions.

The interviews followed a semi-structured guideline comprising three main parts: The first part aimed at learning more about the participants' professional expertise and their experiences with CESS. The second part investigated the interviewees' understanding of ethical problems and paid special attention to their experiences regarding moral distress and moral needs. We used a card sorting approach for the third part to learn more about the participants' concepts of ethical expertise and their expectations of and attitudes toward experts. Card sorting approaches have been shown to be especially suitable for interviews on complex conceptual questions and mappings of ideas (31). The content of the cards was derived from two recent systematic reviews on the outcomes of CESS, which revealed certain connections between the role of an ethics consultant and the outcomes of a consultation (13, 32). Cards were iteratively supplemented with participants' suggestions and included 20 different properties and skills an ethics consultant might be equipped with to fulfill his/her professional role. All participants were invited to rank the importance of the properties or skills on a five-point scale while commenting on their decisions. Interviews were conducted from January 2018 to June 2019 in four different psychiatric hospitals in two different federal states in western Germany.

Non-participant observations were made following a structured observation scheme. The focus was on the question how CESS members fulfilled their role during consultations and how they introduced and described themselves and their role. When possible, the observer also took notes of the CESS members' impressions of the consultation subsequently. All notes were protocolled manually and transcribed within the same day. The cases were observed in two different psychiatric hospitals and a psychiatric long-term care facility in western Germany.

All observations and interviews were carried out by the first author (JH), who has a background in applied ethics, medical ethics and social sciences. Cases to observe were purposively sampled. Participants for interviews were theoretically sampled according to the principles of grounded theory to maximize the variety of the phenomena observed (33). All participants were informed about the study and gave their written informed consent. The study was approved by the research ethics committee of the Medical Faculty of the Ruhr University Bochum (Reg. no.: 17-6194).

### Data Analysis

Interview transcripts and observation protocols were analyzed according to principles of grounded theory following an iterative process of data analysis and data gathering (33). Grounded theory methodologies have proven to be of special advantage in situations where new fields and new questions are addressed and theories of social phenomena (such as the professional role of an ethicist) are investigated (34). Grounded theories enable researchers to gain an in-depth understanding of these phenomena by creating a detailed reconstruction within a shared social horizon of researcher and participants. We deemed this methodology to be suitable insofar as all these criteria apply to our research question.

Preliminary categories were constructed by the main author (JH) based on the first interviews and observations. The initial coding was presented within the research group and discussed during several presentations. These categories served as a basis for the analysis of the remaining material. Finally, all categories were generalized through axial coding. The emerging themes were discussed with several external experts and then presented to national and international experts from the field of clinical ethics consultation or psychiatry and to interview participants on several occasions. Data analysis was used to inform conceptual analysis from a normative perspective. The data analysis was ended after theoretical saturation, that is, the point during the study at which essential changes in the coding by the emergence of new data material was deemed to be unlikely by the researchers.

## Results

A total of 13 interviews with healthcare professionals and CESS members were conducted. In addition, four ethical case consultations were observed. The interviews took an average of 47 min, lasting from 35 to 66 min. The observations lasted from 1.25 to 2.5 h, with an average of 1.6 h. On two occasions, participants in the consultations provided additional information about their impressions in subsequent discussions. Table 1 gives an overview of the interview and observation characteristics.

**Table 1:** Overview of data material.

**Gender**	**Profession**	**Length of interview (min.)**	**Experience with CESS (self-assigned)**
**Study participants (interviews)**			
m	Nursing	53	Low experience
f	Occupational therapy	66	None
f	Psychology/Nursing	45	Some experience
m	Psychiatry	37	None
m	Psychiatry	49	Very experienced
m	Psychiatry	45	None
f	Psychiatry	44	CESS member
f	Psychology	51	CESS member
f	Nursing	51	Some experience
f	Nursing	44	CESS member
f	Nursing	50	CESS member
m	Psychiatry	33	Very experienced
f	Nursing	43	Experienced
**No**.	**Facility**	**Length of observation (h.)**	**Additional notes**
**Observations**			
1	Psychiatric long-term care	2.5	Yes
2	Geriatric psychiatry	1.5	No
3	General psychiatry	1.5	Yes
4	General psychiatry	1.25	No

### Types of Ethical Problems in Psychiatry

Mental health professionals were asked about their experience with ethical problems. We wanted to know what kind of moral needs are raised and what kind of problems mental health professionals want to refer to CESS. It became clear that the identification and description of an ethical problem was in itself a matter for concern. One participant, for example, stated:

And/where ethics. That would have to be defined carefully, in the first place, what it is exactly at all, in everyday life, where ethics and moral aspects play a role, where one does not only talk about, but also some relevancy for further acting can be derived. (Interview 01)

In addition, the observations and interviews showed that this also applied to the results of ethical consultations. Participants of consultations often felt uncertain how to describe and delimit ethical problems. Asked for a rough definition, the participants voiced the impression that ethical problems might be “personal matters” at first and not necessarily an issue on which consensus could be reached due to their subjective nature. A participant explained:

This is a [pausing] this is a subjective evaluation, uhm, and that the question is [for example] when does a patient have permission to go out? Of course, we have psychiatric arguments, too. However, there is a margin and this is often based on morality and we try to discuss it once more. (Interview 07)

It was very important for mental health professionals that CESS members were able to react to this subjective dimension of uncertainty in the problem. All interviewees agreed in the card sorting that analyzing and determining ethical problems might be one of the fundamental features of ethical experts.

**Figure 1:**
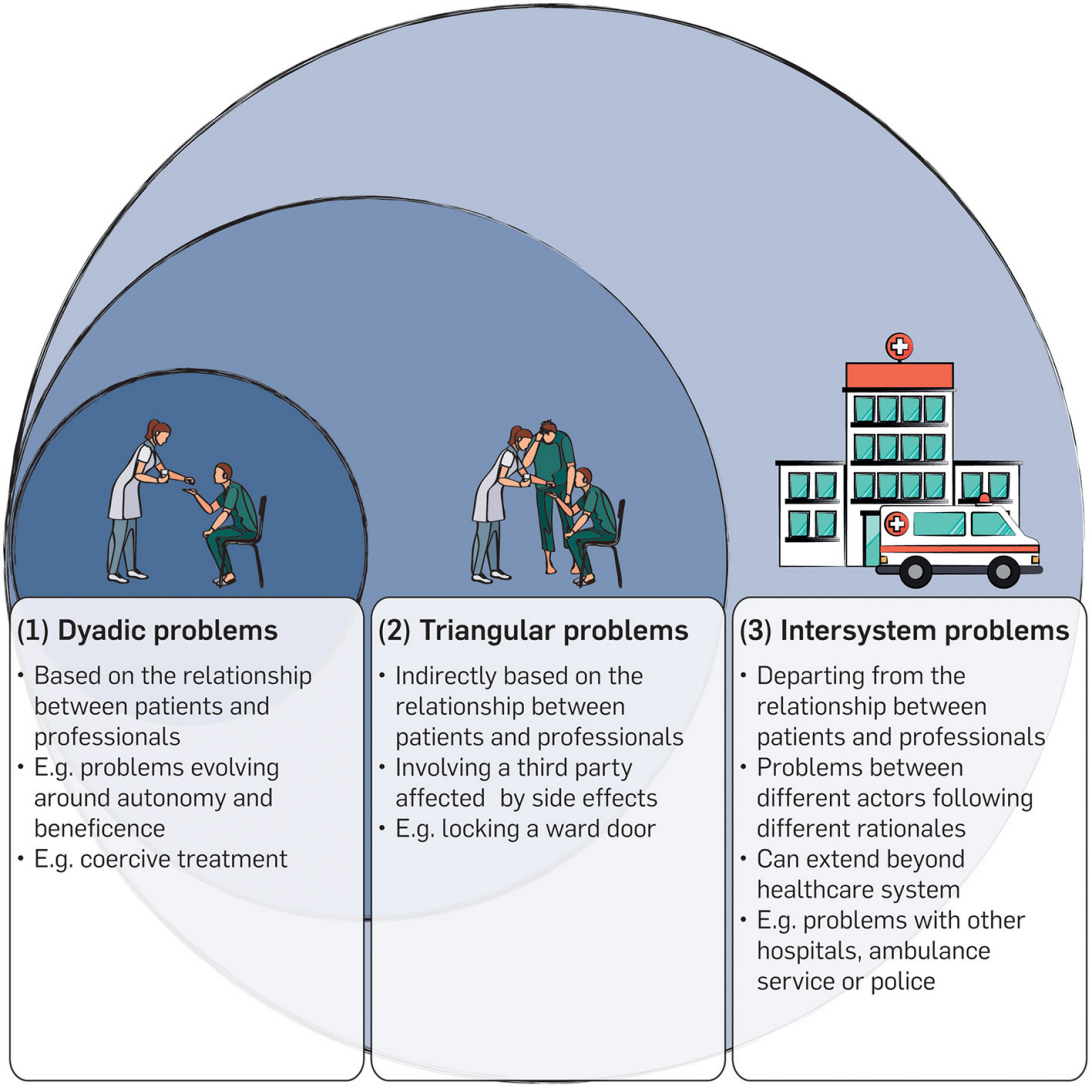
Typology of ethical problems.

Applying a typological perspective relying on the definition of an “ethical problem” as mentioned above, the narratives of the participants finally revealed three different ethical problem types that interviewees wanted to refer to CESS. We called these types (1) “dyadic problems,” (2) “triangular problems” and (3) “intersystem problems.” A graphical representation of these types is given in Figure 1.

(1) Dyadic problems are based on a doctor-patient relationship between individual patients and mental health professionals. A typical example might be the use of coercive measures in situations of self-endangerment. One participant told us:

Well, the first thing that meets the eye is, of course, that we use compulsory treatment, in part. Surely, this is, too, ehm, difficult for patients. Nobody wants that. However, there is no way to get around it, to treat somebody in a compulsory way on the ward, to give medication against his will, to restrain him against his will. (Interview 04)

These problems evolve around the patients' right to self-determination, on the one hand, and the professionals' duty to beneficence, on the other.

(2) Triangular problems often differ from dyadic problems regarding the bearer of the burden, that is, the person whose interests are at stake. One participant used the open-door policy being implemented in his hospital to make clearer what is meant here.

Every locked door implies some sort of violence for professionals and for the patients, because, the fact is, it is about one patient who is coming to the ward, resulting in probably 15 others, who are also in this area which, then, is locked, having to, ehm, live. They have to suffer because they depend on us opening and locking the door. (Interview 01)

In these problems, a third party (i.e., the other patients on the ward) is affected by ethically relevant “side effects” which emerge from a relationship between a patient and mental health professional and, in fact, has nothing to do with them.

(3) Intersystem problems seem to be more complex and are situated on a different level. In contrast to dyadic problems and triangular problems, these ethical problems are neither directly nor indirectly based on the relationship between patients and healthcare professionals. An example of such problems was given in the statement of an interviewee recalling different situations with colleagues from somatic medicine:

So actually, we have really bigger conflicts with other professional groups with our patients if they are somatically ill, and it is about [pausing] An example: A patient needs a transfer to the intensive care unit and there we often find that the medical staff in the general hospital have great reservations about psychiatric patients and we are called again and again to restrain patients mechanically or similarly and that causes friction. Also the topic: The patient is not really stable yet, but he is “psychiatric,” so they try to move him back as soon as possible. (Interview 08)

In another example of this type, an interviewee told us about his concerns about the ambulance service when he was asked which parties usually have stakes in ethical problems. In this case, the implementation of an open-door policy led to patients repeatedly absconding from the ward and making emergency calls to be finally brought back by the rescue services. The participant recalled this situation as an example for the weighing of differing views about ethical obligations:

[That] weighing between different parties involved: treating party, patient, relatives [pausing] ambulance service! Very often. Who might well have claims on us, like, look! “This patient has to be locked up now! You have to end this, now!” (Interview 06)

These problems evolve around different parts of the healthcare system. These different actors (e.g. different wards, hospitals or services outside the institution, such as ambulance services) often follow different rationales concerning the use of different resources to fulfill their purpose. Study participants were severely concerned about problems in which, for example, a different prioritization of resources led to a clash between two or more actors. Regarding the latter quote: While there seem to be ethically justified reasons to adopt an open-door policy to reduce coercion, such an approach might stress the resources of the ambulance services as some patients absconded from the ward and then had to be brought back. As was recalled by the interviewee, the paramedics accused him of an unjustified use of their resources since their necessary reaction prevented them from being available for other (perhaps more urgent) calls at the same time.

These intersystem problems were understood as very demanding by the interviewees. They were connected to deep-rooted concerns. Mental health professionals expressed the need for help, especially in such contexts. Interestingly, perceptions of the CESS members differed. Intersystem problems involving different stakeholders and complex problem schemes sometimes went unnoticed in the CESS members' statements. Some said that these problem types should not be labeled as ethical problems and did not feel responsible for them or were of the opinion that they were not able to deal with such problems. One long-serving CESS member stated:

Yes, well, that would be a little like, mmh. Well, I, yes. It would, well, be a bit of a justice of distribution, or how what amount of resources is occupied by people, which could also be used somewhere else, if you need the ambulance service five times in a situation that does not require somebody to be rescued, right? [pausing] Yes, mmh. Well, in my opinion, the patient always comes first. Of course, it is somehow good if everybody involved feels comfortable or consents to a certain course. But for me, the patient would be in focus and not any claims of paramedics or colleagues in somatic medicine. [pausing] Of course, it might not be that helpful for patients if there is this back and fore or no decision can be made and they are stuck in between. [pausing] I don't know whether this is really an ethical question or an organizational problem. (Interview 11)

### Concepts of Ethical Expertise in Psychiatry

All participants reflected on a strong tension regarding their concept of the role as a “professional ethicist.” This became particularly clear during the card sorting process in the interviews. Many of the participants developed concepts of a tense or contradictory nature. They reflected in detail on the tension between two poles. On the one hand, they described the concept of a neutral and “ethical” person who is equipped with knowledge on the medical context and philosophical theory. According to this, the professional's primary task is to detect and analyze ethical problems. Ethicists should take their own position based on their knowledge and skills. This position was, however, described as not being directive regarding solutions to problems or the ethical content of a problem. One participant stated:

[I also] think that too much is required of an expert to be able to solve an ethical problem, but to recognize and name it, he/she has to be able to do that, but he doesn't have to solve it at all. In turn, however, he/she should be able to make a decision. (Interview 03)

On the other hand, participants expressed their need for ethical guidance. Mental health professionals, especially, described the role as somebody who is able to make better judgements than others. According to this, the ethicists task would be to provide clear recommendations, for example, for the further course of the treatment, and to alleviate the subjective uncertainty, as mentioned above. One interviewee explained:

So, I am the main person responsible for the patient, but I hope that the expert would make a decision of his/her own and that he/she would tell us. (Interview 04)

One participant expressed her expectations regarding the role of a CESS member by comparing it to a situation in court she had experienced some time ago.

I just remembered; I was at law school. Once I was at a court hearing where a judge pronounced a judgement and afterwards, when the students were there, he gave himself time to explain why he had just made that decision. And I found that incredibly helpful, because from the outside, his considerations, which he took into account, that was for me, ehm, I could understand this decision much better and I think maybe in such things the issue simply overlaps a bit. (Interview 03)

According to her understanding, the judge in the example was able to pronounce a judgement due to his function and training, but his efforts to make himself understandable were equally important. In the same way, CESS members were expected to be able to take a clear position and give clear advice. However, participants felt that the authority given to these recommendations depends solely on their ability to make themselves comprehensible.

On the side of the CESS members, this conceptualization led to a situation which was understood as a kind of a dilemma. Participants literally felt torn between the two poles of the concept. On the one hand, they felt that people reach out for their guidance and that it is their responsibility to offer advice in difficult situations. On the other hand, they felt that their matter of expertise, that is, ethics, seems to preclude the idea of outstanding expertise and authoritative guidance. It seems that these people try to master the art of being directive (to offer guidance in difficult cases) without being directive (to offer guidance in an ethical way). This dilemma resulted in very complex and partially contradictory self-descriptions, as in the following case. One CESS member stated:

Am I an ethical expert, surely not. [pausing] But I would say that my experience, and I mean I have been doing this for some years now, it enables me to make somehow better judgments. (Interview 08)

On some occasions, this led to severe frustration because the person did not feel that he/she fulfills both requirements:

Well, I guess we are all rather small-scale ethicists here in medicine, right. (Interview 07)

## Discussion

Successful implementation of CESS in mental healthcare requires interventions and consultants to be able to react to the moral distress of healthcare professionals adequately. As Hem et al. (22) put it, there is a need for qualitative and reflective research to understand how mental health professionals deal with ethical problems and what kind of support might be suitable for them.

Our data reveal three different ethical problem types that can be seen to arise in mental healthcare practice which professionals want to refer to CESS: (1) Dyadic problems, (2) triangular problems and (3) intersystem problems.

Dyadic problems are directly connected to the doctor-patient relationship between individual patients and mental health professionals. They often develop between patient autonomy, on the one hand, and duties to beneficence in medical care, on the other. Although these cases raise serious ethical questions and require careful consideration, our data show that existing structures, including CESS, can deal with them adequately. Cases of this type have, therefore, rarely been a cause for concern in our sample.

Triangular problems also emerge from the relationship between patients and professionals but involve a third party as the bearer of burden whose interest is at stake. Triangular problems pose a certain challenge for the practice of CESS in psychiatry for two reasons. Firstly, these cases are more complex in structure than dyadic problems. There are, for example, more stakeholders whose indirect involvement may be more difficult to assess and evaluate. Secondly, triangular problems raise a need for further theoretical clarification, especially regarding the underlying ethical questions. In many cases, triangular problems are concerned with the fair distribution of burdens. Should, for example, an individual coercive measure (such as seclusion or mechanical restraint) be applied against the will of a person under involuntary commitment who poses a danger to self or to others and repeatedly tries to abscond from an open ward, in order to maintain an open door for all other patients? Such a measure would mean a much stronger restriction of freedom for this individual person while it might benefit others (35). Or vice versa: Do all other patients in this case have to accept a stronger restriction of their freedom than needed in order to minimize the burden for their fellow patient as far as possible? Problems of this kind have rarely been discussed in the literature of ethics in psychiatry. As a consequence, there is a lack of concrete ethical approaches concerning the question of how the burdens could be distributed in an ethically justifiable manner to which CESS could refer.

Intersystem problems describe conflicts between different actors and levels of the healthcare system as a result of the different use of resources, rationales or purposes. This involves complex conflicts that can even extend beyond the healthcare system, including many different stakeholders. Effects emerging from these problems concern health professionals deeply. Intersystem problems seem to result in moral distress, which is not characterized by overarching uncertainty or moral dilemma but by certainty accompanied by constraints. These are typical situations “when one knows the right thing to do, but institutional constraints make it nearly impossible to pursue the right course of action” (36, 37). In terms of content, these problems often reflect the basic tension between psychiatry that is geared toward further opening up and the rather restrictive consequences of society's protective interests. These problems are, therefore, likely to increase in importance in the future as psychiatry continues to develop on its chosen course.

It is notable that CESS members often do not understand this type as an ethical problem or do not feel equipped to deal with it. Problems are not perceived as ethical issues but rather as organizational questions. In addition, CESS members are very reluctant to include this area as part of their ethical responsibility. Different expectations of CESS members and mental health professionals regarding this issue bear a severe risk of misunderstandings and frustration on both sides. However, this finding must be seen against the background that intersystem problems particularly pose great challenges for CESS. Furthermore, it must be argued that, from a conceptual perspective, this type of ethical problem does not correspond to the types of problems in medicine for which CESS was developed originally. These problems are more complex than other types of ethical problems on other levels and clearly depart from the individual relationship between patients and professionals. They include, for example, more stakeholders and institutions from the healthcare sector and can even reach out beyond this system. Including intersystem problems into the responsibility of CESS comes at the price of extensive adaptations in processes and structures. Bringing all perspectives involved to the table and finding solutions on this more systemic level requires high organizational efforts. This includes more sophisticated communication and moderation skills to bridge fundamentally different perspectives and is, doubtless, resource-intensive if it is ever possible at all for simple practical reasons. Moreover, a second point must be added to these pragmatic considerations: Some ethical problems might not even be open for a solution on the level of CESS due to the lack of competencies, power or the addressee of moral complaints. This results in a practical and conceptual challenge for dealing with ethical problems. On the one hand, it is the primary goal of CESS to develop concrete recommendations for ethically justifiable clinical decisions and, thereby, to prevent intersystem problems from being returned to practice as unmanageable. Failure to do so would equal the inability to react to the moral distress of mental health professionals and simply reproduce it on the level of CESS. On the other hand, from a theoretical perspective, recommendations given under such circumstances must be classified as non-optimal moral solutions. They are only justified because the actual problem is out of range. Challenges are, therefore, 2-fold. Firstly, in a practical perspective, ways to communicate this status of advice as non-optimal but justified by circumstances need to be developed. The awareness in CESS members and mental health professionals of the provisionary nature of this advice needs to be raised. Secondly, from a conceptual perspective, structures have to be improved to ensure that intersystem problems can be taken up at higher levels. This is necessary to ensure that implications of ethical problems concerning the systematic level are not left unprocessed once a case on the clinical level has been handled. For these cases, a much stronger network with superior ethical bodies (e.g., at the level of medical associations, or regional or national ethics councils) and policymakers would have to be created. Such a network might be able to counteract the loss of the systematic implications.

Consequently, this would change the nature of CESS significantly compared to their original direct clinical focus. It also requires careful and resource-intensive training of the CESS members and, once again, underlines the importance of ethical expertise as the core of CESS. Our data show a strong inner tension regarding this core of a professional ethicist's role. On the one hand, ethical experts are understood to be neutral people equipped with skills and knowledge to detect and analyze ethical problems. These people's task is to bring together all perspectives on an equal footing. On the other hand, professional ethicists are expected to give advice and guidance and defend ethically justified options in consultations to pave the way for further actions. The CESS members struggle with this role, feeling torn apart between its poles and sometimes even feel unable to satisfy the demands from both sides. Data show considerable inconclusiveness in statements about the professional identities of CESS members. This inconclusiveness of participants' statements mirrors a conceptual problem. As Iltis and Sheehan (29) rightly note, there is a considerable clash between being an expert and exercising advice in the domain of ethics: Expert recommendations give strong reasons to act in accordance with them and experts are mostly appreciated for this guiding force. However, the special character of ethics that assigns moral responsibility to the acting agent seems to preclude giving such authority to others over one's own ethical decisions (29). As a result, CESS members' descriptions of their range of expertise fluctuate between fulfilling the requirement of being an expert in an action-guiding sense and respecting the decision-making capacities and responsibility of those people they counsel.

Although this tension might be ubiquitous in CESS and may never be released completely due to its conceptual nature (38), we suspect its practical emergence to be more typical in mental healthcare. One of the reasons could be the flatter hierarchies in many psychiatric settings. These make it easier to question the status and role of experts and expert knowledge. Another reason could be the relatively small number of case consultations in psychiatry. This implies that there is less experience among professionals and the development of stable professional roles might be still at an early stage. We suggest, however, that this tension needs to be acknowledged as a practical fact that may lead to severe frustration and misunderstanding in CESS members. It should, at least, be mitigated as far as possible in clinical practice to avoid feeling torn apart between the two poles of this professional role. As a starting point, this requires CESS members to be aware of the special difficulties of their expert status and to include constant reflection of one's own position into the training and education of CESS members. A deeper understanding of the typology of ethical problems, as we have developed it here, is an important component to be able to assess the possibilities and the limits of one's own expertise better. It should, therefore, be taken into account in the training and further education of CESS members in psychiatry. A second measure, here, is to emphasize not only the differences and individuality of CESS in psychiatry but also the similarities with somatic medicine. In essence, the question of professional identity concerns all ethical experts. A stronger exchange, for example, through interdepartmental consultations, would contribute to a further development of existing roles on all sides and make it possible to create additional opportunities for exchange and experiences.

## Conclusion

### Strengths and Limitations

Qualitative research enables the exploration of complex social phenomena and underlying assumptions, such as typologies of ethical problems and concepts of expertise, within their native social horizon. In our study, the qualitative data form the basis of a normative and conceptual analysis. This has the strength of ensuring that the theoretical work on concepts is grounded in empirical data and is, hence, both practically relevant and real-world oriented. It creates the opportunity to generate generalizable hypotheses on the challenges highlighted above and sketch ways to improve CESS in psychiatry.

Limitations to be considered in qualitative research concern, *inter alia*, representativity and transferability of the results. It must be stated clearly that results gained in this study cannot be representative due to the qualitative methods applied. Furthermore, the relatively small sample size limits the transferability of our results. In addition, the fact that we gathered our data in the specific German mental healthcare context reduces the scope of our results. We neither want nor are able to make any statement concerning a correlation between a certain institutional or societal context and the occurrence or frequency of any type of ethical problem. These limitations may also influence our normative analysis in terms of the accuracy of the conceptual model as its grade of detail corresponds with the explorative approach of the study. However, this does not limit the applicability of the model itself as a helpful tool for the further advancement of CESS in mental healthcare and especially in psychiatric hospitals.

### Directions of Further Research

Our study warrants further research regarding the ethical problem types in psychiatry and the professional role of an ethicist, putting ethical expertise at its core. The findings concerning the typology of ethical problems show, firstly, a need for research concerning the handling of triangular problems. So far, ethics in psychiatry has devoted comparatively little attention to the question of what an ethically justified distribution of burdens might look like in these cases. Accordingly, there are few points of reference that could help CESS members to translate these difficult theoretical questions into practical counseling processes and ethical recommendations. Secondly, the handling of intersystem problems raises questions concerning the conceptual nature of CESS as an initially clinical intervention that focuses on the relationship between professionals and patients. In mental healthcare – more than elsewhere – CESS members are confronted with ethical challenges that go far beyond this relationship. The fact that this type of ethical problem often cannot be resolved satisfactorily within the limits of CESS and that it can result in non-optimal ethical recommendations can compromise the acceptance of CESS among mental health professionals and the implementation of CESS in mental healthcare institutions. Further research is needed to investigate the quality and quantity of the occurrence of such cases in clinical contexts. In addition, existing strategies of handling and communication need to be reconstructed by means of social science research to generate starting points for the implementation of targeted and practical recommendations for improvements.

Regarding the roles of ethical experts and the professional roles of CESS members in psychiatry, a more systematic development of the professional role of ethics consultants is needed. The role of an ethicist is poorly defined compared to other professional roles in healthcare. However, this is not only because these roles are still at an early stage of development but is due, above all, to the special nature of ethical expertise, which seems to differ from other forms of expertise. There are currently only a few approaches in the theoretical literature to a consistent conceptualization of the specific roles of ethics consultants, which urgently need to be developed further.

## Data Availability Statement

The raw data supporting the conclusions of this article will be made available by the authors, without undue reservation.

## Ethics Statement

The studies involving human participants were reviewed and approved by Research Ethics Committee of the Medical Faculty of the Ruhr University Bochum. The participants provided their informed consent to participate in this study.

## Author Contributions

JH, JG, JS, and JV designed this study. JH, JG, and GJ contributed to the implementation of the research. JH carried out the field work and the data analysis with input from JG, JS, GJ, and JV. JH wrote the first version of the manuscript. All authors contributed to and approved its final version.

## Conflict of Interest

The authors declare that the research was conducted in the absence of any commercial or financial relationships that could be construed as a potential conflict of interest.
